# Molecular Topology as Novel Strategy for Discovery of Drugs with Aβ Lowering and Anti-Aggregation Dual Activities for Alzheimer’s Disease

**DOI:** 10.1371/journal.pone.0092750

**Published:** 2014-03-26

**Authors:** Jun Wang, David Land, Kenjiro Ono, Jorge Galvez, Wei Zhao, Prashant Vempati, John W. Steele, Alice Cheng, Masahito Yamada, Samara Levine, Paolo Mazzola, Giulio M. Pasinetti

**Affiliations:** 1 Department of Neurology, Mount Sinai School of Medicine, New York, New York, United States of America; 2 Medisyn Technologies, Inc. Minnetonka, Minnesota, United States of America; 3 Department of Neurology and Neurobiology of Aging, Kanazawa University, Kanazawa, Japan; 4 Molecular Topology & Drug Design Unit, University of Valencia, Valencia, Spain; 5 Geriatric Research, Education and Clinical Center, James J. Peters Veterans Affairs Medical Center, Bronx, New York New York, United States of America; Bioinformatics Institute, Singapore

## Abstract

**Background and Purpose:**

In this study, we demonstrate the use of Molecular topology (MT) in an Alzheimer’s disease (AD) drug discovery program. MT uses and expands upon the principles governing the molecular connectivity theory of numerically characterizing molecular structures, in the present case, active anti-AD drugs/agents, using topological descriptors to build models. Topological characterization has been shown to embody sufficient molecular information to provide strong correlation to therapeutic efficacy.

**Experimental Approach:**

We used MT to include multiple bioactive properties that allows for the identification of multi-functional single agent compounds, in this case, the dual functions of β-amyloid (Aβ) -lowering and anti-oligomerization. Using this technology, we identified and designed novel compounds in chemical classes unrelated to current anti-AD agents that exert dual Aβ lowering and anti-Aβ oligomerization activities in animal models of AD. AD is a multifaceted disease with different pathological features.

**Conclusion and Implications:**

Our study, for the first time, demonstrated that MT can provide novel strategy for discovering drugs with Aβ lowering and anti-aggregation dual activities for AD.

## Introduction

Alzheimer’s disease (AD) is by far the most prevalent neurodegenerative disease of aging with an estimated prevalence of 5.3 million in the U.S. growing to 11–16 million by the year 2050 [1]; [2]. AD is a major cause of functional disability in older persons and causes massive costs for society and caregivers. Estimated direct health care costs for AD in the U.S. is $148 billion annually, with an additional $94 billion in unpaid costs to caregivers [1]; [2]. Given the increasingly aged structure of the population and the impacts of AD care on healthcare costs in an era of impending health care reform, it is important to find treatments that reduce the burden and cost of the disease.

We do not yet have a definitive treatment for AD. There are several novel AD treatments currently in trials, largely based on the amyloid hypothesis, with the target mechanism of reducing brain amyloid load. Unfortunately, several of these agents have recently produced null results, including Alzhemed (a fibrillization inhibitor), Flurizan (an allosteric modulator of **γ**-secretase), Dimebon, RAGE inhibitor, and a gamma-secretase inhibitor. There are two anti-amyloid antibody therapies (i.e., passive immunization) currently in phase III trials (bapineuzamab and solaneuzamab), of which one (bapineuzamab) has not demonstrated significant clinical effects in a phase II trial. It is becoming increasingly evident that more effective treatments of established AD need to be developed, and there is an urgent need to explore novel strategies for AD treatments.

The major hypothesis of the pathogenesis of AD is the amyloid hypothesis: increasing content of Aβ peptides lead to the formation of insoluble Aβ fibrillar aggregates, which are the major constituents of senile plaques associated with neuronal loss in AD [3]. The amyloid hypothesis is supported by substantial genetic [4] and preclinical evidence [5] including treatment effects of passive immunotherapies in mouse models of AD [6]. However, there is controversy regarding details of the hypothesis and the optimal molecular target for intervention [7]. Furthermore, there is a poor correlation between plaque number and cognitive dysfunction in AD [8], demonstrated by the observation that many older persons die cognitively intact with significant brain amyloid burden (particularly diffuse plaques) [9]. Furthermore, there is evidence of neuronal dysfunction (evidenced by decreased glucose uptake imaged with FDG-PET) in early AD prior to neuronal loss or plaque deposition in the temporal and parietal cortex [10]. Hence, there is evidence for neuronal dysfunction well before neuronal loss in the absence of plaque deposition. This neuronal dysfunction may be mediated by soluble oligomeric forms of Aβ. Low-n Aβoligomers (from dimmers to octamers) are found in AD brains [7]; [11] and have been shown to decrease long-term potentiation (LTP) in mouse models of AD *both in vitro* and *in vivo* [12]; [13]. These low-n Aβ oligomers are powerful synaptoxins and thus potential targets for new treatments of AD [7]. Agents that reverse oligomerization of synthetic Aβ peptides have been demonstrated to reverse the inhibition of LTP [14].

Molecular topology is an application of graph theory, built upon physical, biological, and chemical properties, to create a classification function to predict the activity of specified candidate molecules *in *vivo and *in vitro.* Molecular topology (MT) topological indices (TIs) have been used as a basis for the development of the Forward Engineering platform, to identify novel chemical entities for drug discovery. Model development is an iterative process that allows for flexibility and refinement of parameters, targets, and specificity in real-time. The process does not require a specific mechanism of action or receptor-ligand interactions, as in traditional QSAR methods.

In our study, Forward Engineering is used to identify and/or design novel compounds in chemical classes unrelated to current anti-AD agents with dual anti-amyloid/anti-aggregation activity to enable (1) prevention and/or reduction of Aβ peptides, (2) halting Aβ deposition and/or (3) reduction of soluble Aβ1–42. This multifaceted approach targets early steps in the pathogenesis of AD that are also believed responsible for clinical progression of the disease [15]; [16]. MT allows for the inclusion of multiple bioactive properties, thus the potential identification of multi-functional single agent compounds. The Forward Engineering platform uses and expands upon the principles governing molecular connectivity theory of numerically characterizing molecular structure, or in the present case, active anti-AD drugs/agents using topological descriptors to build each model. Topological characterization has been shown to embody sufficient molecular information to provide strong correlation to therapeutic efficacy. This characterization method is necessary because molecular topology (MT) is founded on the assumption that (1) molecular formulas can be mathematically characterized and (2) mathematically determined parameters of molecules can be correlated with the molecules’ experimentally measured properties (e.g., IC50 values) [17]–[19].

Hence, the most important tools to identify the molecular signature of a compound are the topological indices (TIs) derived from procedures (algorithms) for converting the topological structure of a molecule into a single characteristic number. The core features of Forward Engineering are speed, low cost, risk mitigation absorption, distribution, metabolism, excretion & toxicity (ADME/Tox) evaluation, and novel compound discovery [20]; [21].

In this study, we set to test as proof of concept, the use of Forward Engineering platform to screen molecules that may possess both Aβ -lowering and anti-oligomerization activities for preventing and/or treating AD.

## Methods

### Reagents

FDA approved drugs and natural compounds were obtained from MicroSource Discovery Systems Inc (Gaylordsville, CT). Chemical compounds were purchased from Sigma Aldrich (St. Louis, MO), Ryan Scientific (Mt. Pleasant, SC), Apollo Chemicals (Burlington, NC), Nacalai USA, Inc (San Diego, CA), Aurora Fine Chemicals (San Diego, CA), Indofine chemical Company (Hillsborough, NJ) and Toronto Research Chemicals (Toronto, Canada).

### Molecular Topology Model

To form good training sets, current/experimental agents with desired activity, e.g. Aβ lowering activity or anti-aggregation activity, were assembled. Compounds included as part of each training set were carefully selected after analyzing and discussing the type of assays that were used to characterize the compound. Each training set included a very heterogeneous group of structurally diverse Aβ lowering and/or anti-aggregation agents and a group of inactive compounds that were structurally similar to the active Aβ lowering and/or anti-aggregation agents with respect to atoms, heteroatoms, bonds, and cycles. Next, each compound was associated with %Aβ lowering or anti-aggregation activity, and the corresponding 2-D formula. This information was used as input data for topological characterization. After associating activity with 2-D formulae, each compound was classified on the basis of inactive/active prior to uploading into Forward Engineering.

Thereafter, the 2-D structure (or molecular formula) of anti-AD agents/drugs associated with bioactivity were uploaded into Forward Engineering. Mathematically characterization of these agents/drugs in terms of (1) the number of atoms, (2) how many other atoms each is connected to within the molecule, (3) and whether the atoms are connected to form a straight chain with branches, rings or combinations thereof was initiated. Next, the steps required to build each model included:

1. Selecting relevant TIs for the training set of compounds using Forward Engineering [17].
2. Characterizing the compounds with the TI’s to convert the data into well-defined topological sets of numerical values [22]–[24].
3. Mapping the TIs to compounds to create a discriminant function (model) between the numerical values and the analyzed anti-AD activity based on a linear discriminant analysis (LDA) [20]; [25].
4. Cross-validating analysis to make sure that the model works and is “good” by seeing if the model was able to discern activity after chemical database screening and/or screening a database [18]

### Forward Engineering

**Figure 1:**
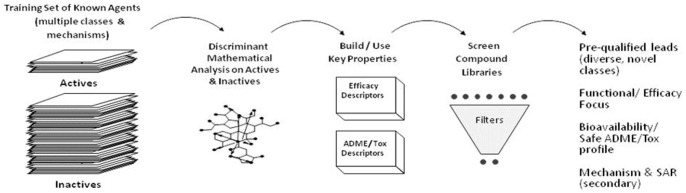
Forward Engineering process schematic. Each model iteration consists of five steps: (1) compilation of a training set of active and inactive compounds with associated data, (2) mathematical translation of structure into TIs, (3) use of TIs to develop model descriptors, (4) compound libraries are screened using the model filters to identify compounds that fit the desired properties, (5) selection of pre-qualified lead compounds for evaluation.

A simplified scheme of Forward Engineering is presented in Figure 1.

### In silico Screening

We screened 1600 FDA approved drugs (Microsource Discovery System Inc, CT) for Aβ-lowering activity using primary neurons from Tg2576 mice (see below). We also screened 720 natural compounds (Microsource Discovery System Inc, CT) for anti-oligomerization, using *in vitro* aggregation assay (see below). These data were used to build the molecular topology models with signature of desired properties (Aβ-lowering and anti-oligomerization dual function). The models were then used to screen both synthetic and/or natural bioactive databases, including the American Chemical Database (ACD), Screening Compound Database (SCD), Dictionary of Natural Products (DNP) and Isolates of Natural Products (INP), which have a combined listing of over 25 million compounds including over 300,000 natural compounds. Thereafter, an output set of compounds containing known actives, inactives, and novel synthetic and natural bioactives is generated. Each compound includes a predicted efficacy in the same units specified for training set compounds to provide an estimate of predicted potency.

### In vitro Screening of Compound for Aβ Lowering Activity in Primary Neurons

Embryonic-day (E)16 cortico-hippocampal neuronal cultures were prepared from heterozygous Tg2576 transgenic mice as previously described [26]. Compounds were dissolved in DMSO as 10 mM stock. Primary neurons prepared in 96-well plates were treated with 0.1 μM, 1 μM, 10 μM, 50 μM, and 100 μM of each drug in duplicate for ∼16 hours and conditioned medium was collected for Aβ detection. Cell viability was assessed using a commercial available LDH assay kit according to the manufacture’s instruction (Promega). Losartan, an angiotensin receptor blocker that reduces Aβ, and trandolapril, an angiotensin converting enzyme inhibitor that increases Aβ, were used in each round of Aβ-lowering screening.

### In vitro Aβ_1–40_ and Aβ_1–42_ Aggregation Assay

Aβ_1–40_ and Aβ_1–42_ peptides were purchased from American Peptide (Sunnyvale, CA). Peptides were solubilized in HFIP (Sigma), and dried overnight [27]. Compound stock was dissolved in phosphate buffer (pH 7.4, Invitrogen) at 200 μM. Aβ_1–40_ and Aβ_1–42_ (100 μg/ml in PBS) were mixed with different concentration of compound at 1∶1 volume and incubated at 37°C for 24 hours. The effect of compound on Aβ aggregation was analyzed by western blot analysis using 6E10 antibody. Two β-adrenergic receptor blockers, Carvedilol, which strongly inhibits Aβ aggregation, and Acebutolol, which has no effect on Aβ aggregation, were used as positive and negative controls in the aggregation assay.

### Photo-induced Cross-linking of Unmodified Proteins (PICUP) Assay

PICUP assay was performed as previously described [28]–[30]. Briefly, freshly isolated LMW (low molecular weight) Aβ_1–42_ or Aβ_1–40_ peptide was mixed with 1 (“x1”), 2 (“x2”), 5 (“x5”) or 10 (“x10”) mM tris(2,2′-bipyridyl)dichlororuthenium(II) (Ru(bpy)) and ammonium persulfate (APS) in the presence or absence of testing compound in 10 mM phosphate, pH 7.4. The mixture was irradiated for 1 second, and quenched immediately with Tricine sample buffer containing 5% β-mercaptoethanol [28]. The reaction was subjected to SDS-PAGE and visualized by silver staining (SilverXpress, Invitrogen).

### *In vivo* Testing

Female Tg2576 transgenic mice expressing the human 695-amino acid isoform of the APP containing the Swedish double mutation (APPswe), [(APP695) Lys670→Asn, Met671→Leu] driven by a hamster prior promoter were used in this study [31]. For short-term feasibility study, mice were treated with 2 mg/kg/day of the candidate compound delivered through their drinking water. The treatment started at 6 months of age, prior to the development of AD-type amyloid neuropathology. Following one month treatment, the animals were sacrificed and the levels of soluble Aβ oligomer content, as well as total Aβ content in the brain, were examined. All procedures and protocols were approved by the Mount Sinai School of Medicine’s Institutional Animal Care and Use Committee (IACUC) through the Center for Comparative Medicine and Surgery.

### Amyloid Peptides Measurements

Total Aβ_1–40_ or Aβ_1–42_ in the brain or from the conditioned medium were quantified by sandwich ELISA (Invitrogen), as previously described [32]. Soluble Aβ oligomers were isolated and measured as previously prescribed, with some modifications [30]. Specifically, the detergent-soluble proteins were extracted with extraction buffer (50 mM Tris-HCl, pH 7.4; 150 mM NaCl; 1 mM EGTA; 3% SDS; 0.5% Triton X-100; 1% deoxycholate; 0.1 mM PMSF, 0.2 mM 1,10-phenanthroline monohydrate; protease inhibitor cocktail; and phosphatase inhibitor cocktails), mechanically homogenized and centrifuged at 4°C, 13,000 rpm for 90 minutes and the supernatant contains soluble oligomeric Aβ. The supernatant was subsequently depleted of endogenous immunoglobulins by sequential incubation with Protein A and Protein G Sepharose 4 FF resin. The supernate was then analyzed by commercially available ELISA kit that specifically detects aggregated β-amyloid using protocols provided by the manufacturer (Invitrogen).

### Statistics

Effectiveness of test compounds will be determined using statistical analysis. Differences between two groups will be analyzed by a two-tailed t-test. Analysis of variance will be used to compare three or more groups, and Bonferroni’s multiple comparisons test will be used to detect differences across multiple groups.

## Results

### Identification of the 8 Lead Compounds

**Figure 2:**
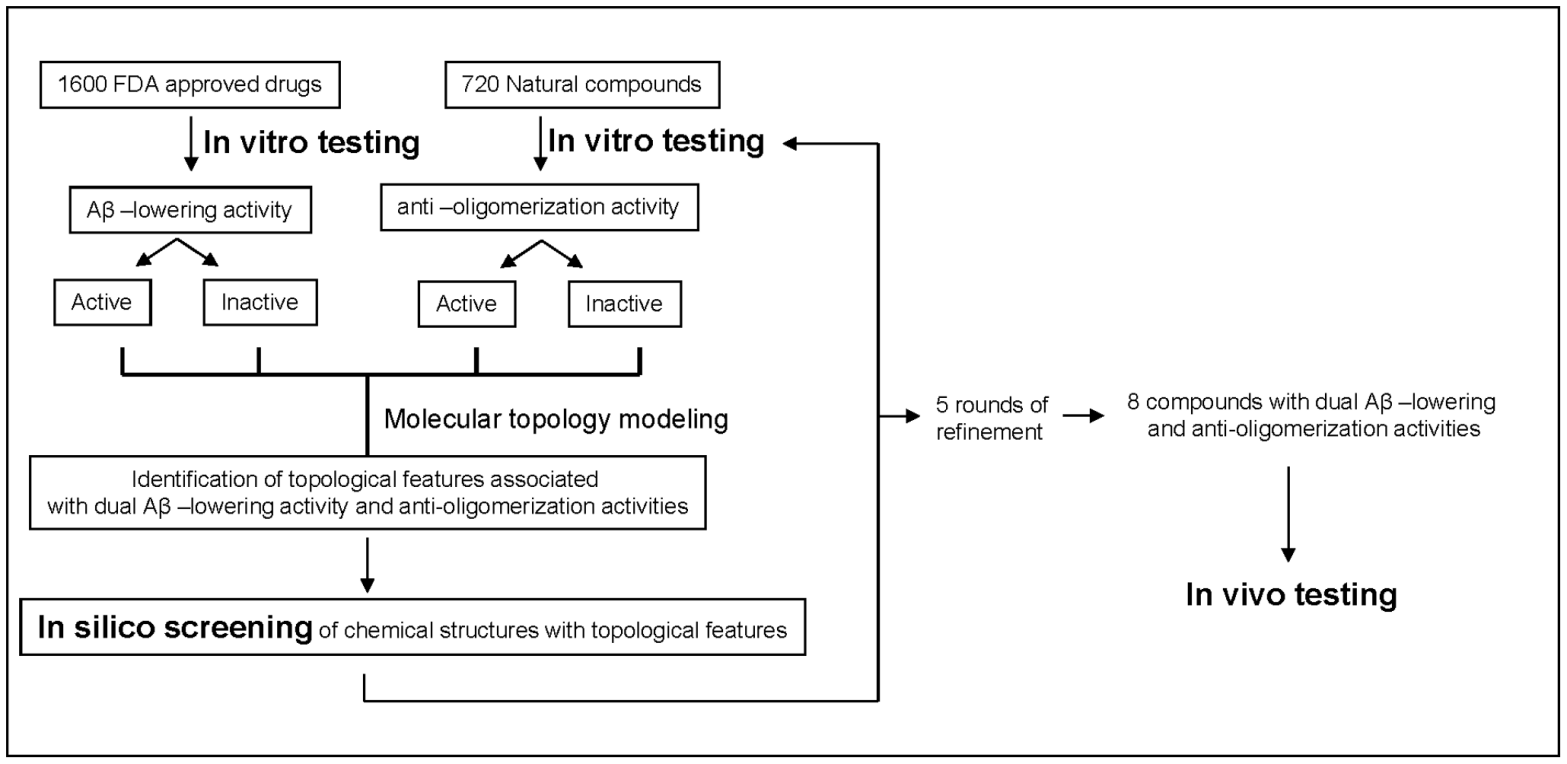
Schematic drug discovery procedure using molecular topology modeling.

**Figure 3:**
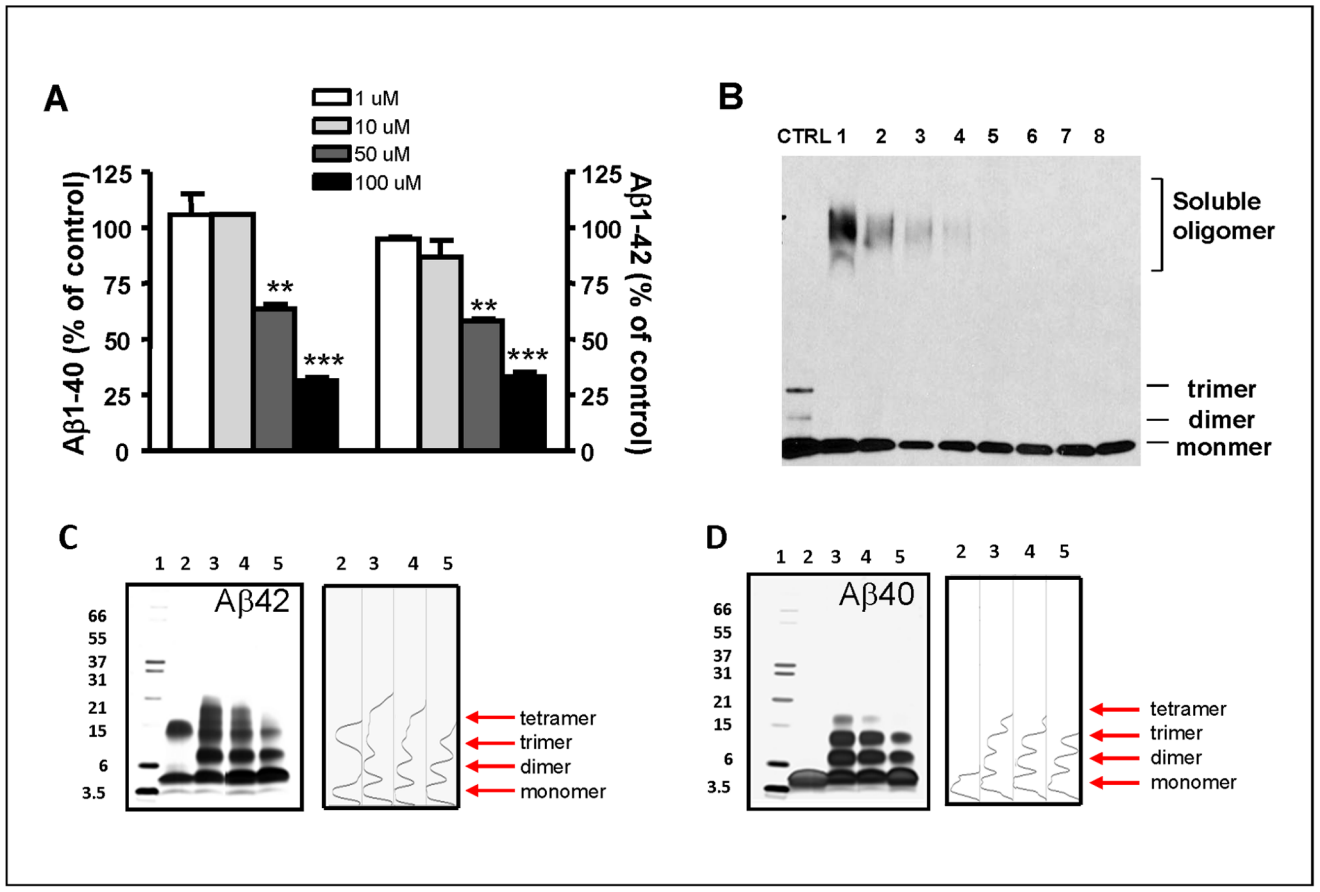
Representative *in vitro* screening results for Aβ -lowering and anti-oligomerization activity. (A) Measurement of Aβ_1–42_ and Aβ_1–40_ in conditioned medium from Tg2576 primary neurons treated with different concentration of testing compound (B) Dose dependent inhibition of testing compound on synthetic Aβ_1–42_ peptides aggregation into HMW oligomeric Aβ forms. Aβ_1–42_ (final concentration 10 μM) was incubated with various concentrations of compound at 37°C for 24 hours. Bands were visualized by western blot analysis probed with 6E10 antibody. MW: molecular weight, CTRL is Aβ_1–42_ without incubation, Lane 1–8, 0, 10, 20, 40, 80,160, 320 and 640 μM of compound. (C and D) SDS-PAGE of Aβ_1–42_ (C) and Aβ_1–40_ (D) in the presence or absence of testing compound following PICUP. Aβ_1–42_ or Aβ_1–40_ was cross-linked in the presence or absence of testing compound and the bands were visualized using silver staining. Lanes 1: molecular weight, lane 2: Aβ_1–42_ or Aβ_1–40_ peptides without PICUP reaction; lane 3: Aβ_1–42_ or Aβ_1–40_ peptides following PICUP reaction; Lanes 4 and 5: PICUP of Aβ peptides in the presence of an equimolar concentration (lane 4) or 10x molar excess (lane 5) of testing compound.

The screening scheme is presented in Figure 2. Screening of the 1600 FDA approved drugs identified 800 drugs that could lower Aβ by 10%, among which, 184 lowered Aβ by 30%. We also found 241 drugs that could increase Aβ by 10%, among which 26 drugs increased Aβ by 30%. These data were used to build the first MT model followed by the addition of screen results of anti-aggregation activity from 720 natural compounds. A combined list of over 25 million compounds including over 300,000 extracts were screened *in silico* against the topological features. Compounds with desired Aβ-lowering and anti-aggregation characteristics from the *in silico* screening were chosen for *in vitro* testing. *In vitro* testing included Aβ-lowering activity in primary neuron culture, anti-aggregation activity by in vitro aggregation assay followed by confirmation by PICUP assay. Figure 3 shows typical data from a representative compound that exhibit both Aβ-lowering activity and anti-aggregation activity. Figure 3A shows a dose response reduction of Aβ_1–40_ and Aβ_1–42_ in primary neurons isolated from Tg2576 mice following overnight incubation with the testing compound. Figure 3B shows a dose response reduction of high molecular weight (HMW) soluble oligomer formation in the presence of testing compound, the reduction of HMW soluble oligomers is accompanied by the increase concentration of monomers (Figure 3B). The anti-aggregation activity was then confirmed by the PICUP assay that examines initial peptide-to-peptide interactions that are necessary for spontaneous oligomerization of Aβ peptides [33] (Figure 3C and 3D). In the absence of cross-linking, only Aβ_1–42_ trimers and monomers and Aβ_1–40_ monomers were revealed on the SDS-PAGE gel (Lane 2, figure 3C for Aβ_1–42_ and 3D for Aβ_1–40_, please note the Aβ_1–42_ trimer band is an SDS-induced artifact). Using cross-linking to stabilize Aβ peptide-to-peptide interactions, we confirmed that Aβ peptides spontaneously aggregate into multimeric conformers; Aβ_1–42_ formed a mixture of monomers and oligomers of orders 2–6, whereas Aβ_1–40_ formed a mixture of monomers, dimers, trimers and tetramers (Figure 3C and 3D, lane 3). We found that incubation of Aβ_1–42_ with testing compound at equimolar concentrations significantly reduced the formation of oligomers of orders 3–6, and at 10x access, almost completely blocked the generation of n>3 oligomers and significantly reduced the generation of dimers and trimers (Figure 3C, lanes 4 and 5). Similarly, testing compound significantly reduced the amount of Aβ_1–40_ tetramer at equimolar concentrations, completely abolished the formation of aggregated Aβ_1–40_ tetramer and significantly reduced the generation of dimer and trimer species at 10x access molar concentration (Figure 3D, lanes 4 and 5). Densitometry quantitations of the amount of oligomers are presented in right panels of Figures 3C and 3D.

**Figure 4:**
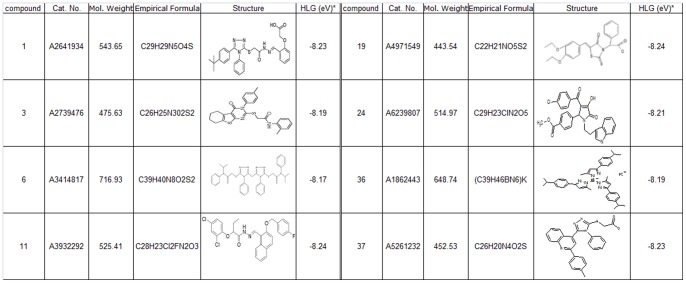
Molecular name, database identifier, empirical formula, 2-D structure, and topological indexes of HOMO and LUMO Gap (HLG) of the eight compounds that are able to exert dual Aβ -lowering and anti-oligomerization by *in vitro* testing.

The data resulted from the *in vitro* testing were fed to the MT for further refinements, followed by another round of *in silico* screening and *in vitro* testing. A total of 5 rounds of refinements were made and a total of 131 compounds were screened in vitro: first round: 30 compounds; second round: 32 compounds; Third round: 24 compounds; fourth and fifth rounds: 34 compounds and 11 compounds respectively. Following 5 rounds of refinements (Figure 2), we identified 8 lead compounds exerting potent concentration-dependent Aβ-lowering and anti-aggregation dual function *in vitro* (Figure 4 and Table S1).

### Identification of Lead Compound with in vivo Efficacy

Following *in vitro* identification of the 8 lead compounds, we tested the effect of oral administration of these compounds on AD-type neuropathology in transgenic mouse models of AD following a one-month short-term treatment regime. We found that the drug treatment was well tolerated as reflected by their general health indexes, such as normal food and water intakes, normal body weight (Table 1) and normal grooming of the mice. Following the short-term treatment, animals were sacrificed for brain and plasma amyloid analysis. We found that compound 1, 3, 19 and 36 could significantly reduce the brain levels of soluble Aβ as well as total Aβ (Table 1), while compound 24 significantly reduced the brain level of soluble Aβ without influencing the total Aβ content in the brain (Table 1). In parallel studies, we confirmed that the drug treatment did not influence the expression of the APP transgene in the brain (data not shown).

**Table 1:** Short-term feasibility studies of the 8 lead compounds in reducing Aβ neuropathology in Tg2576 mouse model of AD.

compound	body weight	Oligomer in the brain	Total Aβ in the brain
I.D.	(% of control post treatment)	(% of control)	(% of control)
**CTRL**			
**(veh. Treated)**	100.0±2.1	100.0±5.9	100±21.8
**1**	95.5±4.4	79.3±5.9*	72.9±4.7*
**3**	96.1±4.2	78.8±5.6*	72.1±2.1*
**6**	92.6±1.5	102.0±9.9	87.8±21.9
**11**	91.6±2.9	100.1±4.2	123.2±22.6
**19**	95.6±2.4	82.6±8.6*	56.4±9.4*
**36**	101.5±5.6	82.7±3.5*	41.3±4.5*
**24**	98.7±1.2	79.3±6.6*	96.2±19.4
**37**	97.7±3.5	88.2±9.1	126.1±34.0

Tg2576 mice were treated with 2 mg/kg/day of the candidate compound delivered through their drinking water for 4 weeks, Body weight, brain neuropathology including oligomeric Aβ as well as total brain Aβ were compared to vehicle-treated control Tg2576 mice (n = 5 per group, *p<0.05).

## Discussion

AD is an age-associated neurological disorder posing tremendous burden on the US health system. Currently there is no cure. The available treatments only improve disease symptoms and have not demonstrated significant beneficial effects on AD. There is a growing consensus that the failure to develop an effective intervention for AD may be due, in part, to the fact that almost all past preclinical and clinical trials have been designed to target individual pathological features.

Forward Engineering uses pre-existing data in conjunction with molecular structure to develop a mathematical characterization of the desired functional outcome, based on a specifically defined set of biological properties and/or therapeutic targets. This approach allows for custom integration of positive attributes as well as developing screening functions to weed out negative attributes, such as side effects seen in current therapies. By identifying the most promising functional chemical signatures and properties that lead to failure, the platform can develop a pipeline of pre-qualified new drug candidates. Forward Engineering represents a new cost-effective, materials efficient method for pre-qualifying compounds for preclinical efficacy studies.

Topological indices can be used to screen both synthetic and natural bioactive databases to identify compounds that have a molecular signature that is similar to the ideal defined by the desired functional outcome, such as Aβ-lowering and anti-aggregation activities. The process allows for the inclusion of multiple bioactive properties, such as toxicity and brain penetration. Thus, multi-functional single-agent compounds can be identified. In this proof of concept study, through five rounds of refinements, we identified 8 compounds that possess Aβ-lowering and anti-aggregation dual activities when tested in vitro. In vivo short-term dosing studies revealed that 4 out of the 8 compounds can simultaneously reduce oligomeric Aβ as well as total Aβ in the brains of APP transgenic model of AD.

One of the advantages of MT in drug discovery is that it can help improve the success/failure ratio by reducing the guesswork in the process. Based on our study and previous experience, active compound selection success rates can range up to 85% based on novel compound activity alone. This rate is greater than High Throughput Screening rates of 0.1%. AD is multifactorial and involves several different etiopathogenic mechanisms. The other advantage of MT is that it can prioritize multiple features when designing molecules. In AD, these features include the anti-hyperphosphorylation, anti-aggregation properties for microtubule-binding protein tau, and anti-inflammatory qualities. Future drug discovery using MT can include these disease-modifying activities, which might lead to the identification of compounds that can simultaneously target multiple pathological features of AD.

## Supporting Information

Table S1 **Aβ-lowering activity and anti-aggregation activity of the 8 lead compounds.** Data presented for Ab-lowering is based on 100 uM testing compound in primary neurons derived from Tg2576 mice and anti-aggregation is based on equal molar ratio of compound and Ab (see material and methods for experiment details).
(PPTX)
