# Supplementary material for: Molecular Topology as Novel Strategy for Discovery of Drugs with Aβ Lowering and Anti-Aggregation Dual Activities for Alzheimer’s Disease
Source: PLoS One. 2014 Mar 26;9(3):e92750. doi: 10.1371/journal.pone.0092750 (PMC3966818; doi:10.1371/journal.pone.0092750)
Supplement: Table S1 — Aβ-lowering activity and anti-aggregation activity of the 8 lead compounds. Data presented for Ab-lowering is based on 100 uM testing compound in primary neurons derived from Tg2576 mice and anti-aggregation is based on equal molar ratio of compound and Ab (see material and methods for experiment details). (PPTX) [file pone.0092750.s001.pptx]

## Slide 1
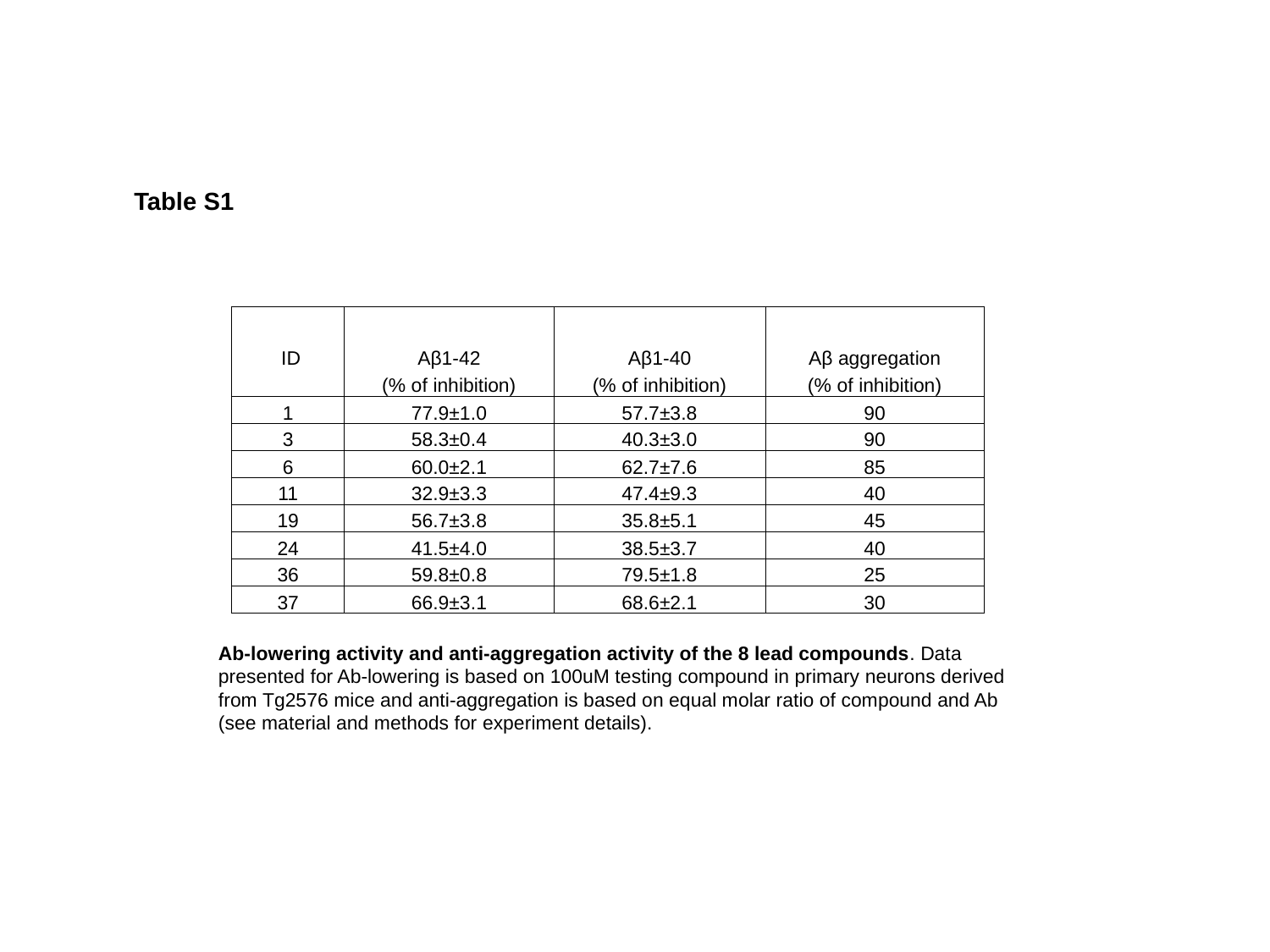

Table S1
Ab-lowering activity and anti-aggregation activity of the 8 lead compounds. Data presented for Ab-lowering is based on 100uM testing compound in primary neurons derived from Tg2576 mice and anti-aggregation is based on equal molar ratio of compound and Ab (see material and methods for experiment details).
| ID | Aβ1-42 | Aβ1-40 | Aβ aggregation |
| --- | --- | --- | --- |
| | (% of inhibition) | (% of inhibition) | (% of inhibition) |
| 1 | 77.9±1.0 | 57.7±3.8 | 90 |
| 3 | 58.3±0.4 | 40.3±3.0 | 90 |
| 6 | 60.0±2.1 | 62.7±7.6 | 85 |
| 11 | 32.9±3.3 | 47.4±9.3 | 40 |
| 19 | 56.7±3.8 | 35.8±5.1 | 45 |
| 24 | 41.5±4.0 | 38.5±3.7 | 40 |
| 36 | 59.8±0.8 | 79.5±1.8 | 25 |
| 37 | 66.9±3.1 | 68.6±2.1 | 30 |
